# Solvothermal Synthesis of Multiple Dihydropyrimidinones at a Time as Inhibitors of Eg5

**DOI:** 10.3390/molecules26071925

**Published:** 2021-03-30

**Authors:** Xiao-Qiang Jiang, Shi-Quan Chen, Yan-Fei Liu, Xin-Guang Pan, Dan Chen, Shi-Fan Wang

**Affiliations:** 1 Department of Pharmacy, School of Life and Pharmaceutical Sciences, Hainan University, Haikou 570228, China or xqjiang2021@163.com (X.-Q.J.); breezysmile.c.s.q@163.com (S.-Q.C.); 18868816346@163.com (Y.-F.L.); PanXinGuang898@163.com (X.-G.P.); chen18662023423@163.com (D.C.); 2 Dongfang Municipal Bureau of Agriculture and Rural Affairs, Dongfang 572600, China

**Keywords:** solvothermal synthesis, dihydropyrimidinones, Eg5 inhibitor

## Abstract

Solvothermal synthesis of multiple dihydropyrimidinones at a time has been developed in inexpensive and green bio-based solvent lactic acid without any additional catalysts or additives. By this method, thirty new dihydropyrimidinone derivatives were synthesized in two batches and characterized. All of the compounds were screened by Eg5 motor protein ATPase assay, and the positive compounds were tested against the Caco-2 cell line, HeLa cell line, L929 cell line and T24 cell line in vitro. Among them, compound C9 exhibited the best inhibitory activity against motor protein ATPase with an IC_50_ value of 30.25 μM and significant cytotoxic activity in the micromolar range against the cells above. The Lineweaver–Burk plot revealed that compound C9 was a mixed-type Eg5 inhibitor. A molecular modeling study using the Discovery Studio program was performed, where compound C9 exhibited good binding interaction with Eg5 motor protein ATPase, and this was consistent with the attained experimental results.

## 1. Introduction

**Scheme 1:**
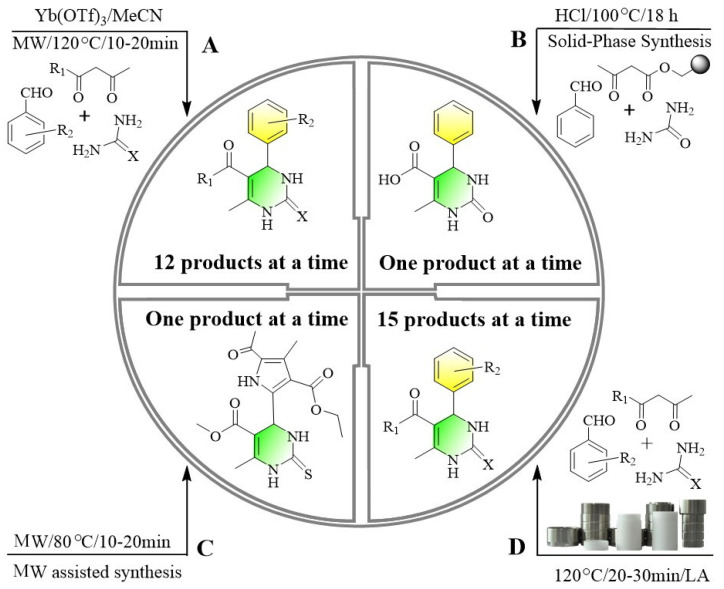
(**A**) Microwave-assisted synthesis of 12 DHPM derivatives at a time in MeCN. (**B**) Solid phase synthesis of DHPM derivatives obtaining one product at a time. (**C**) Microwave-assisted synthesis of DHPM derivatives obtaining one product at a time. (**D**) Solvothermal synthesis of 15 diversely functionalized DHPM derivatives at a time in lactic acid.

Colchicine, vinca alkaloids, taxanes and epithiolone are currently used in cancer treatment. They specifically inhibit the function of microtubules, which form the principal structure of the mitotic spindle [1,2,3,4,5,6]. However, because microtubules play essential roles in the maintenance of cytoskeleton functions and intracellular transport processes, the microtubule-binding chemotherapeutics also exhibit several serious side effects, such as neutropenia, peripheral neuropathy and severe myelosuppression [7,8]. Compared to the traditional microtubule-binding drugs, inhibitors of microtubule-associated proteins, such as the mitotic motors (kinesins), may hopefully lead to cell-cycle arrest and ultimately to apoptosis but with fewer side effects. Mitotic kinesins participate in the regulation of cellular replication and formation of the bipolar mitotic spindle [9]. Eg5 (also known as KIF11/KSP/kindle spindle protein) is a member of the kinesin superfamily and is over expressed in proliferating tissues and the majority of tumors [10]. In 1999, monastrol, a kind of dihydropyrimidinone (DHPM), was identified in a phenotype-based screen as the first Eg5-specific inhibitor [11]. It was discovered that monastrol is an allosteric inhibitor and binds to the motor domain of Eg5 (amino acids 1-368) [12,13]. The great potential of DHPMs in pharmaceutical fields has accordingly triggered growing interest in their synthetic study [14]. In 1893, Italian chemist Pietro Biginelli reported on the acid-catalyzed three-component condensation reaction, which is well known as the Biginelli reaction. Recently, a large number of optimized procedures have been reported, such as microwave-assisted synthesis (Scheme 1A) [15,16,17], solid phase synthesis (Scheme 1B) [18] and ultrasound-assisted synthesis (Scheme 1C) [19]. However, each method has certain restrictions with regard to scope and reaction conditions, for example costs of synthesis, unrecoverable catalysts, strong acidic conditions, used of toxic organic solvents, long reaction times, low yields and difficult work up [20,21,22,23].

Solvothermal synthesis has been widely used in the preparation of many inorganic materials [24,25,26,27,28], but there are few reports on the solvothermal synthesis of organic compounds or drugs [29]. Recently, when we studied the solvothermal synthesis of DHPMs in lactic acid medium, we were surprised to find that the Biginelli reaction is very effective (Scheme 1D). Herein, we wish to report solvothermal synthesis of 15 diversely functionalized DHPM derivatives at a time as inhibitors of Eg5.

## 2. Results

### 2.1. Chemistry

**Scheme 2:**
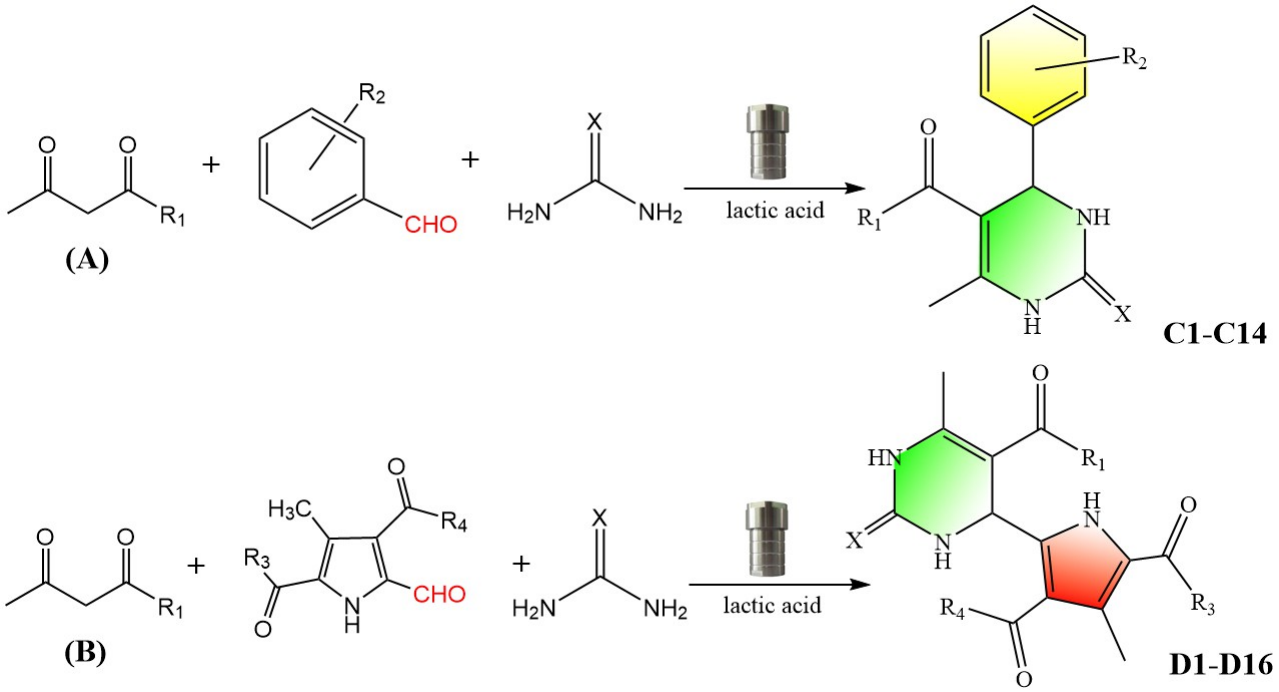
Structural diversity in the DHPM scaffolds was generated by employing nine 1,3-dicarbonyl compounds, nine benzaldehyde derivatives and urea/thiourea. (**A**) Route for solvothermal synthesis of dihydropyrimidinones containing a benzene ring (**C1**–**C14**). (**B**) Route for solvothermal synthesis of dihydropyrimidinones containing a pyrrole ring (**D1**–**D16**).

To optimize the reaction conditions, different reaction temperatures, times and the amount of lactic acid were investigated in the solvothermal synthesis of DHPMs. If benzaldehyde derivatives are applied for the synthesis of DHPMs containing benzene ring (Scheme 2A), the time and temperature parameters can be applied, as given in Table 1. If pyrrolaldehyde derivatives are applied for the synthesis of DHPMs containing a pyrrole ring (Scheme 2B), the time and temperature parameters can be applied, as given in Table 2. Compared to conventional heating methods, a special physical and chemical environment is provided under high temperature and high pressure of solvothermal conditions. Due to the fact that the solvothermal synthesis is carried out in a uniform system, the product has better dispersibility, higher purity and better crystallinity than other methods. In most cases, the products are isolated by simple filtration from water and crystallized from the hot mixed solvent of ethanol and water without any cumbersome post-processing methods. Another important feature of solvothermal synthesis is the ability to protect the existence of a variety of functional groups during the reaction, such as ester, nitro and halides, etc. Owing to these facts, it is not surprising that this method is likely to be used in organic synthesis for both lead optimization and lead generation.

### 2.2. Identification of Potential Eg5 Motor Protein Inhibitors by Screening In Vitro Figures, Tables and Schemes

**Figure 1:**
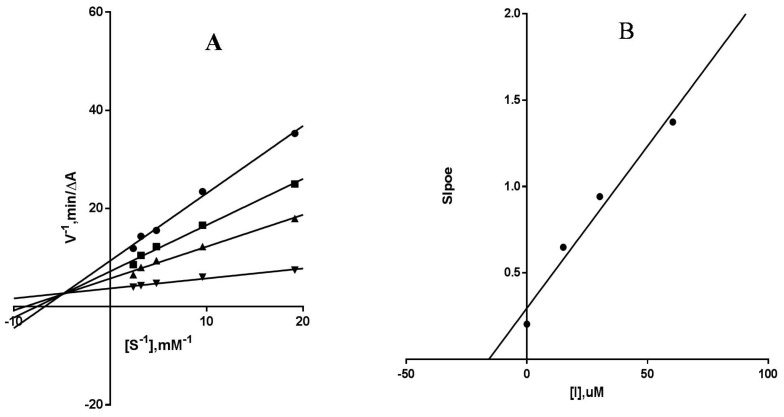
Steady-state inhibition of Eg5 by **C9**. (**A**) Lineweaver–Burk plot of reciprocal of initial velocities versus reciprocal of five fixed ATP concentrations (0.417 mM, 0.313 mM, 0.209 mM, 0.104 mM and 0.052 mM) in the absence (▼) and presence of **C9** at 15.13 μM (▲), 30.25 μM (■), 60.50 μM (●). Lines represent a curve fit of the global data set to a mixed-type inhibition model. (**B**) Secondary plots of the Lineweaver–Burk plot, slope versus various concentration of **C9**, with Ki = 15.73 μM.

All of the 30 compounds and the positive control, monastrol, were tested for inhibition of Eg5 motor protein with ATPase activity assay as described below. **C9**, **D1**, **D2**, **D3**, **D4**, **D7**, **D10**, **D12** and **D14** showed activities in micromolar range concentrations (Table 3). The Lineweaver–Burk plot (Figure 1) revealed that compound **C9** was a mixed-type Eg5 inhibitor, with Ki = 15.73 μM.

### 2.3. Cytotoxity of MTS Assay

Compounds **C9**, **D1**, **D4**, **D7**, **D10** and **D14** showed superior growth inhibition of the Caco-2 cell compared to monastrol (IC_50_ = 111.62 μM), with IC_50_ in a range of 65.8–107.57 μM. Moreover, **C9**, **D1**, **D3** and **D10** showed growth inhibition of the Hela cell line with IC_50_ in a range of 75.59–127.99 μM (monastrol IC_50_ > 150 μM). Furthermore, **C9**, **D1**, **D4** displayed moderate activity against the T24 cell line, with IC_50_ in a range of 103.21–130.21 μM. However, compounds **C9**, **D1**, **D4**, **D7**, **D12** and **D14** also exerted cytotoxic effect on the L929 cell, which is a kind of ordinary fibroblast, but not cancer cells (Table 3). The results suggest that among these nine compounds, compound **C9** and **D1** showed significantly better inhibition against proliferation of the four cell lines in a dose dependent manner at micromolar range concentrations.

### 2.4. Molecular Docking Simulations

**Figure 2:**
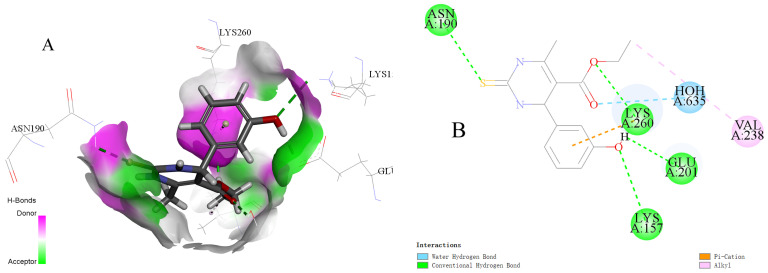
(**A**) The 3D view of the binding mode between the positive control monastrol and kinesin Eg5; the pink and green zones represent the hydrogen bond interactions. (**B**) The schematic diagram generated using the 2D diagram of Discovery Studio 4.1 shows the interactions between the positive control monastrol and the neighboring residues of kinesin Eg5.

**Figure 3:**
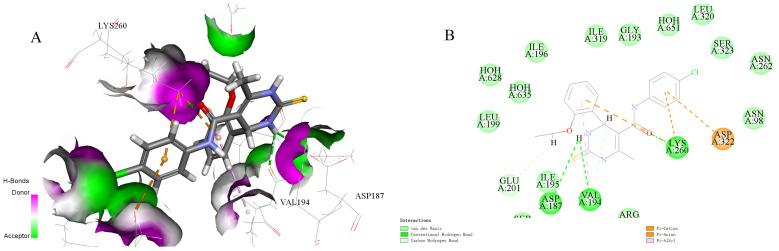
(**A**) The 3D view of the binding mode between the most potent compound **C9** and kinesin Eg5; the pink and green zones represent the hydrogen bond interactions. (**B**) The schematic diagram generated using the 2D diagram of Discovery Studio 4.1 shows the interactions between the most potent compound **C9** and the neighboring residues of kinesin Eg5.

The results of molecular docking simulations showed that both monastrol and C9 could strongly bind to Eg5 protein. The binding mode of monastrol and kinesin Eg5 is shown in Figure 2. The binding mode of compound **C9** and kinesin Eg5 is shown in Figure 3.

## 3. Discussion

The Biginelli reaction has been successfully conducted under various acidic conditions [30,31,32], such as Bronsted acids [33] and Lewis acids [34,35,36]. However, it is rarely found that the Biginelli reaction is carried out under the solvothermal condition of lactic acid. It is well known that lactic acid (2-hydroxy propionic acid) is a natural organic acid, and in earlier research, we have already reported that lactic acid is a very useful green solvent to promote some synthesis reactions [37]. During this study, special attention was paid to the development of diversely functionalized DHPM derivatives at a time as inhibitors of Eg5. In the subsequent experiment, we found that the Biginelli reaction was carried out very efficiently under solvothermal conditions in lactic acid, which was proved to be simple to operate with good yield. To optimize the reaction conditions, different amounts of lactic acid for the solvothermal synthesis of DHPM derivatives were also explored (Table 4), and the optimum amount of lactic acid was 2 to 4 mL at 2 mM starting reactants.

A wide variation of β-dicarbonyl compounds and aldehydes were chosen in order to explore the impact of different substituents on the development of the diversely functionalized DHPM derivatives. We found that β-diketone, β-ketoacid ester and β-ketoamide are all effective dicarbonyl reagents for the Biginelli reaction under solvothermal conditions in lactic acid. The electronic effect of substituents on the aromatic ring was also observed. In most cases, aromatic aldehydes containing electron-donating groups gave higher yields in shorter times than did aromatic aldehydes containing electron-withdrawing groups. If thiourea is used instead of urea, the temperature of Biginelli reaction under solvothermal conditions will be increased by about 10 °C. We also found that it is useful to use excessive urea components, and the optimal ratio of aldehydes, 1,3-dicarbonyl compounds and urea/thiourea is 1:1:1.2. By a simple aftertreatment procedure of washing with water and recrystallization, the corresponding pure DHPM derivatives were obtained and characterized by IR, ^1^H NMR and ESI-MS.

The bioassay results indicated that the oxypyrimidinone compounds **C1**–**C8** containing an aromatic benzene ring had almost no inhibitory activity, but the thiopyrimidinones with an aromatic benzene ring showed a certain inhibitory effect. Among them, **C9** showed better inhibitory activity with IC_50_ of 30.25 μM (IC_50_ of the positive control monastrol is 51.74 μM). On the contrary, when the aromatic benzene ring was replaced by a pyrrole ring, the oxypyrimidinones of **D1**, **D2**, **D3**, **D4**, **D7** and **D10** showed activities in micromolar range concentrations (Table 3).

To further elucidate the interaction between the positive control monastrol, the most potent compound **C9** and kinesin Eg5, molecular docking was performed using CDOCKER protocol in Discovery Studio 4.1. The results suggested that both of the positive control monastrol and the most potent compound **C9** could bind tightly with the kinesin Eg5 (Figure 2 and Figure 3). Figure 2A shows that a binding pocket was formed to hold the positive control monastrol. Moreover, Figure 2B shows that monastrol binds with four hydrogen bonds of Asn190, Lys260, Glu201 and Lys157, as well as one alkyl repulsive force of Val280 in kinesin Eg5. Figure 3A,B show that compound **C9** was mainly surrounded by Asp187, Ile195, Leu199, Ile196, Ile319, Gly193, Leu320, Ser323, Asn262, Asn98, Asp322, Lys260 and Val194, which are active amino acids for the ligand–receptor of kinesin Eg5 interaction. As expected, the acyl oxygen atom and the secondary amino hydrogen atom at the ring of hydropyrimidinone formed three essential hydrogen bonds with Asp187, Lys260 and Val194 in kinesin Eg5, respectively. Moreover, compound **C9** also has potential aromatic interaction with Asp322. In addition, residues Ile195, Leu199, Ile196, Ile319, Gly193, Leu320, Ser323, Asn262 and Asn98 in the proximity of the ligand were unfavorable to van der Waals forces. These results revealed that compound **C9** had a better binding ability to kinesin Eg5 than did the positive control monastrol, and this was consistent with the attained experimental results.

## 4. Materials and Methods

### 4.1. Chemistry

#### 4.1.1. General Procedure

All reagents are A.R. grade and purchased from Aladdin Industrial (Shanghai, China) and used without any additional purification. All reactions were run under an air atmosphere. Melting points (°C, uncorrected) were determined on a XT4 MP apparatus (Taike Corp, Beijing, China). The IR spectra were recorded on a Thermo iS10 IR spectrometer (Shanghai, China) (KBr pellets) and ^1^H NMR spectra were recorded in CDCl_3_ or d^6^-DMSO on a Bruker DPX400 spectrometer (Beijing, China) with TMS and solvent signals allotted as internal standards. Splitting patterns are as follows: *s*, singlet; *d*, doublet; *dd*, double doublets; *t*, triplet; *q*, quartet; *m*, multiplet. Chemical shifts are reported in δ (ppm) and coupling constants are given in Hertz. ESI mass spectra were recorded on the Shimadzu LCMS-IT-TOF mass spectrometer (Shanghai, China). All the products were identified by comparison of their physical and spectral data with those of authentic samples.

#### 4.1.2. General Procedure

Two millimole of aldehyde, 2 millimole of 1,3-dicarbonyl compound, 2.4 millimole of urea or thiourea and 2.0–4.0 mL lactic acid were well mixed in sealed reactors at room temperature. Then the sealed reactors were heated in an electrothermal drying oven at 100–120 °C for 50 min (urea) or 120–130 °C for 80 min (thiourea). The reaction mixture was poured into water, filtered and recrystallized from ethanol to give compounds **C1**–**C14** or **D1**–**D16**. IR spectra, ^1^H NMR spectra and the data of synthesized compounds C1–C14 and D1–D16 were shown in Supplementary Materials Figures S1–S60.

### 4.2. Inhibition of Eg5 Motor Protein ATPase Activity

#### 4.2.1. Preparation of Eg5 Motor Protein

Eg5 motor domain (amino acids 1-368) was cloned into pET22b and expressed in *Escherichia coli* BL21 (DE3) cells. It was purified over His-tag affinity and gel-filtration chromatography, dialyzed against storage buffer (150 mM NaCl, 20 mM Tris, pH 8.0) and stored at −20 °C.

#### 4.2.2. Preparation of Tubulin

Tubulin was purified from pig brain, as described in the literature [38], and stored at −60 °C in BRB80 (80 mM K-Pipes, pH 6.8, 1 mM EGTA, 1 mM MgCl_2_). Before the assay of Eg5 ATPase, microtubules were assembled in the presence of paclitaxel and were then separated from unassembled tubulin by centrifugation as described in the literature [39].

#### 4.2.3. Measurement of ATPase Rates

Inhibitory activities of the compounds were measured using a microtubule-activated ATPase assay. The compounds, paclitaxel stabilized microtubules, Eg5 motor protein and BRB80 were added to wells of a 96-well plate in a total reaction volume of 38.5 μL. Reactions were started by the addition of 522.11 μM ATP in a total reaction volume of 98.5 μL, incubated at 37 °C for 30 min and terminated by the addition of 15 μL of perchloric acid. The mixture was centrifuged (10,000 rpm) 10 min at room temperature. The supernatant from this centrifugation step was mixed with HCl 1 M, 33 mM malachite green, 775 mM ammonium molybdate tetrahydrate. After 10 min the absorbance was measured at 630 nm using a microplate reader (BIO-RAD iMark^TM^). Control of DMSO was used at a final concentration of 2.5%, a control experiment at this DMSO concentration showed no effect on the microtubule-activated ATPase activity. Monastrol was used as the positive control. All conditions were performed in three independent duplicate experiments and averaged data points are shown as mean values ± SD, IC_50_ values were calculated with SPSS17.0 software. The primary data were shown in Supplementary Materials Tables S1–S3.

#### 4.2.4. Lineweaver–Burk Plot Analysis

Assays followed methods similar to that described above for the primary Eg5 motor protein ATPase assay. For either study, compound **C9** were tested at 60.50 μM, 30.25 μM, 15.13 μM, 0 μM. ATP concentration was varied from 52 μM to 417 μM. The enzyme concentration tested was 100 nM. All conditions were performed in three independent duplicate experiments. The data were analyzed with GraphPad Prism 6.02 software.

### 4.3. Cytotoxicity of MTS Assay

The positive compounds (IC_50_ < 100 μM) obtained from the primary Eg5 motor protein ATPase assay were tested for antiproliferative activity in vitro against Caco-2 cell line, Hela cell line, L929 cell line and T24 cell line by performing MTS assay. Cells were introduced into 96-well plates at a density of 1000 cells per well. After 24 h incubation, the cells were exposed with compounds at final concentrations ranging from 0.5 to 100 µM for 24 h. DMSO was used at a final concentration of 0.025% as blank control, monastrol was used as the positive control. Then 20 µL (CellTiter 96^®^ AQueous One Solution Promega, Madison, WI, USA) reagent was added in each well. After 2 h at 37 °C in a 80% humidified, 5% CO_2_ atmosphere, the absorbance at 490 nm was recorded using a microplate reader (BIO-RAD iMark^TM^). The results were obtained in three independent duplicate experiments. The primary data were shown in Supplementary Materials Tables S4–S7. The IC_50_ values for these compounds were calculated from a non-linear regression graph plotted between the percentage of cell viability and concentration with SPSS17.0 software.

### 4.4. Molecular Modeling and Docking Studies

Crystal structure of human kinesin Eg5 with a new monastrol-based inhibitor (R)-mon97 binding (PDB ID 2IEH) was downloaded from RCSB Protein Data Bank (http://www.rcsb.org/pdb). The receptor and the ligands were pretreated. For receptor (2IEH) preparation, water molecules were removed, the hydrogen atoms were added and partial charges were assigned based on the CHARMm force field. For ligand preparation, 3D structures of the ligands were generated in Discovery Studio 4.1 and then energy minimization was performed by the CHARMm force field. Finally, the semiflexible (the receptor was rigid, whereas the ligands were flexible during the docking process) docking mode CDOCKER was used to run the docking program in Discovery Studio 4.1 (Laboratory of CADD, Hainan University, China) with the binding sites (coordinates x, 38.4271, y, 14.1162 and z, 100.177) and receptor radius of 11.4 Å. After molecular docking, conformation on the lowest CDOCKER interaction energy pose was selected as the most probable binding conformation and the docking results were studied with CDOCKER energy scores, CDOCKER interaction energy scores, hydrogen bond interaction and the binding mode pattern.

## 5. Conclusions

In summary, we developed an efficient and eco-friendly method for the solvothermal synthesis of multi dihydropyrimidinone derivatives at a time. Thirty new compounds were synthesized and screened for inhibition of Eg5 motor protein. Compound **C9** showed more activity than monastrol. The Lineweaver–Burk plot revealed that compound **C9** was a mixed-type Eg5 inhibitor. In the cell viability assay, compound **C9** displayed significant cytotoxic activity in the micromolar range against the Caco-2 cell line, HeLa cell line, T24 cell line and L929 cell line. A molecular modeling study using the Discovery Studio program was performed, where compound **C9** exhibited good binding interaction with Eg5 protein, and this was consistent with the attained experimental results.

## Figures and Tables

**Table 1:** Optimization of reaction conditions for solvothermal synthesis of dihydropyrimidinones (**C1**–**C14**) ^a^.

Entry	R_1_	R_2_	X	Time (min)	Temp. (°C)	LA (mL)	Yield (%)
**C1**	C_6_H_5_NH	2-C_2_H_5_O	O	50	100	2.0	82
**C2**	2-CH_3_C_6_H_4_NH	2-C_2_H_5_O	O	50	100	2.0	81
**C3**	2-CH_3_OC_6_H_4_NH	2-C_2_H_5_O	O	50	100	2.0	83
**C4**	4-ClC_6_H_4_NH	2-C_2_H_5_O	O	50	100	2.0	85
**C5**	4-ClC_6_H_4_NH	2-CH_3_O	O	50	100	2.0	76
**C6**	C_6_H_5_NH	2-OH	O	50	100	2.0	76
**C7**	(CH_3_)_3_CO	2-C_2_H_5_O	O	50	100	2.0	78
**C8**	2-CH_3_C_6_H_4_NH	2-NO_2_	O	50	100	2.0	73
**C9**	4-ClC_6_H_4_NH	2-C_2_H_5_O	S	80	120	3.0	82
**C10**	2-CH_3_C_6_H_4_NH	2-C_2_H_5_O	S	80	120	3.0	79
**C11**	C_6_H_5_NH	2-C_2_H_5_O	S	80	120	3.0	80
**C12**	C_6_H_5_NH	3,4-(CH_3_O)_2_	S	80	120	3.0	84
**C13**	4-ClC_6_H_4_NH	3,4-(CH_3_O)_2_	S	80	120	3.0	82
**C14**	2-CH_3_C_6_H_4_NH	2-NO_2_	S	80	120	3.0	71

^a^ The amounts of starting material, aldehydes (2 mM), 1,3-dicarbonyl compounds (2 mM), urea/thiourea (2.4 mM), in lactic acid (LA) medium.

**Table 2:** Optimization of reaction conditions for solvothermal synthesis of dihydropyrimidinones (**D1**–**D16**) ^a^.

Entry	R_1_	R_3_	R_4_	X	Time (min)	Temp. (˚C)	LA (mL)	Yield (%)
**D1**	2-CH_3_OC_6_H_4_NH	CH_3_O	C_2_H_5_O	O	50	120	4.0	80
**D2**	2-CH_3_C_6_H_4_NH	CH_3_O	C_2_H_5_O	O	50	120	4.0	79
**D3**	2-CH_3_OC_6_H_4_NH	C_2_H_5_O	CH_3_O	O	50	120	4.0	81
**D4**	2-CH_3_C_6_H_4_NH	C_2_H_5_O	CH_3_O	O	50	120	4.0	78
**D5**	2-CH_3_C_6_H_4_NH	C_2_H_5_O	C_2_H_5_O	O	50	120	4.0	80
**D6**	2-CH_3_C_6_H_4_NH	CH_3_O	CH_3_O	O	50	120	4.0	75
**D7**	4-ClC_6_H_4_NH	C_2_H_5_O	C_2_H_5_O	O	50	120	4.0	84
**D8**	4-ClC_6_H_4_NH	C_2_H_5_O	CH_3_O	O	50	120	4.0	80
**D9**	C_6_H_5_NH	CH_3_O	CH_3_O	O	80	120	4.0	76
**D10**	C_6_H_5_NH	CH_3_O	C_2_H_5_O	O	80	120	4.0	79
**D11**	C_6_H_5_NH	C_2_H_5_O	C_2_H_5_O	O	80	120	4.0	81
**D12**	CH_3_O	C_2_H_5_O	C_2_H_5_O	S	80	130	4.0	78
**D13**	CH_3_CH_2_O	CH_3_O	CH_3_O	S	80	130	4.0	80
**D14**	C_6_H_5_	CH_3_O	CH_3_O	S	80	130	4.0	71
**D15**	C_6_H_5_	CH_3_O	C_2_H_5_O	S	80	130	4.0	70
**D16**	C_6_H_5_NH	CH_3_O	C_2_H_5_O	S	80	130	4.0	81

^a^ The amounts of starting material, aldehydes (2 mM), 1,3-dicarbonyl compounds (2 mM), urea/thiourea (2.4 mM), in lactic acid (LA) medium.

**Table 3:** IC_50_ values for inhibition of microtubule-stimulated KSP ATPase activity and mitotic arrest in cell-based assays for compounds.

Entry	ATPase Activity ^a^	Caco-2 Cell ^a^	Hela Cell ^a^	T24 Cell ^a^	L929 Cell ^a^
**C9**	30.25	65.80	75.59	103.21	85.97
**D1**	38.73	107.57	127.99	130.43	124.87
**D2**	67.29	116.48	neg	neg	neg
**D3**	53.57	neg	114.85	neg	neg
**D4**	71.29	80.54	neg	128.54	94.54
**D7**	63.25	81.94	neg	neg	94.69
**D10**	64.36	107.53	107.65	neg	neg
**D12**	57.37	147.55	neg	neg	121.26
**D14**	71.86	102.77	neg	neg	136.93
**Monastrol**	51.74	111.62	neg	129.14	neg

^a^ neg, negative (IC_50_ > 150 μM).

**Table 4:** Influence of different amounts of lactic acid on the solvothermal synthesis of DHPM derivatives ^a^.

Entry	LA 1 mL/Yield (%)	LA 2 mL/Yield (%)	LA 3 mL/Yield (%)	LA 4 mL/Yield (%)	LA 5 mL/Yield (%)
**C1**	65	82	80	74	68
**C8**	61	73	69	65	61
**C9**	69	77	82	74	71
**C14**	58	66	71	68	62
**D1**	69	74	78	80	75
**D9**	61	67	72	76	70
**D16**	63	71	78	81	76

^a^ The amounts of starting material, aldehydes (2 mM), 1,3-dicarbonyl compounds (2 mM), urea/thiourea (2.4 mM), in lactic acid (LA) medium.

## Data Availability

All the data is contained within the article and Supplementary Material. Any further information presented in this study is available on request from the corresponding author.

## Supplementary Materials

The following are available online, Figures S1–S60: IR Spectra, ^1^H NMR spectra and the data of synthesized compounds **C1**–**C14** and **D1**–**D16**. Tables S1–S3: The data of potential Eg5 motor protein inhibitors identification. Tables S4–S7: The data of cytotoxicity of MTS assay.

## Sample Availability

Samples of the compounds are available from the authors but can only be availed after consent from all the authors.
