# Supplementary material for: Solvothermal Synthesis of Multiple Dihydropyrimidinones at a Time as Inhibitors of Eg5
Source: Molecules. 2021 Mar 30;26(7):1925. doi: 10.3390/molecules26071925 (PMC8037182; doi:10.3390/molecules26071925)
Supplement: Supplementary file 1 [file molecules-26-01925-s001.zip › Supplementary Materials No.1.doc]

Solvothermal synthesis of multiple dihydropyrimidinones at a time as inhibitors of Eg5

*Compound* **C1**

Crystallized from the hot mixed solvent of ethanol and water (v:v=10:1) to obtain crystals in colorless needles, mp 243-245 ˚C; IR(KBr)cm^-1^: 3439.34, 3311.28, 3106.26, 2971.75, 2353.63, 1713.10, 1671.46, 1636.23, 1594.60, 1431.26, 755.50; ^1^H NMR (400MHz, DMSO-d^6^) δ=9.56 (s, 1H, CONH), 8.64 (s,1H, NH on the pyrimidine ring), 7.56 (s, 1H, NH on the pyrimidine ring), 7.25-7.54 (m, 5H, H on the benzene ring), 7.21-7.23 (m, 4H, H on the benzene ring), 5.64 (s, 1H, CH), 3.91-3.99 (q, 2H, CH_2_CH_3_), 2.00 (s, 3H, CH_3_ on the pyrimidine ring), 1.25 (t, 3H, CH_2_CH_3_; ESI-MS calcd. For C_20_H_21_N_3_O_3_, m/z 351.1583, found 350.1512 [M-H]^-^.

**Figure S1**. IR spectra of synthesized compound **C1**

**Figure S2**. ^1^HNMR spectra of synthesized compound **C1**

*Compound* **C2**

Crystallized from the hot mixed solvent of ethanol and water (v:v=10:1) to obtain crystals in colorless needles, mp 251-253˚C; IR (KBr) cm^-1^: 3442.54, 3272.80, 3106.26, 2978.15, 2923.71, 1713.10, 1668.26, 1620.22, 1508.13, 1450.48, 752.29; ^1^H NMR (400 MHz, DMSO-d^6^) δ = 8.87 (s, 1H, CONH), 8.64 (s, 1H, NH on the pyrimidine ring), 7.25 (s, 1H, NH on the pyrimidine ring), 7.17-6.99 (m, 8H, H on the benzene ring), 5.66 (s, 1H, CH), 4.00-3.95 (q, 2H, CH_2_CH_3_), 2.11 (s, 3H, CH_3_ on the pyrimidine ring), 1.87 (s, 3H, CH_3_ on the benzene ring), 1.30 (t, 3H, CH_2_CH_3_); ESI-MS calcd. For C_21_H_23_N_3_O_3_, m/z: 365.1739, found 364.1663 [M-H]^-^.

**Figure S3**. IR spectra of synthesized compound **C2**

**Figure S4**. ^1^HNMR spectra of synthesized compound **C2**

*Compound* **C3**

Crystallized from the hot mixed solvent of ethanol and water (v:v=10:1) to obtain crystals in colorless needles, mp 263-265˚C; IR(KBr)cm^-1^: 3420.12, 3349.67, 3234.37, 3135.09, 2971.75, 2939.72, 2898.09, 1697.08, 1665.06, 1597.80, 1520.94, 1460.09, 752.29; ^1^HNMR (400 MHz, DMSO-d^6^) δ=8.87 (s, 1H, CONH), 8.04 (s, 1H, NH on the pyrimidine ring), 7.90 (s, 1H, NH on the pyrimidine ring), 6.93-7.28 (m, 8H, H on the benzene ring), 5.54 (s, 1H, CH), 4.02-4.07 (q, 2H, CH_2_CH_3_), 3.65 (s, 3H, OCH_3_), 2.19 (s, 3H, CH_3_ on the pyrimidine ring), 1.32 (t, 3H, CH_2_CH_3_); ESI-MS calcd. For C_21_H_23_N_3_O_4_, m/z: 381.1689, found 380.1614 [M-H]^-^.

**Figure S5**. IR spectra of synthesized compound **C3**

**Figure S6**. ^1^HNMR spectra of synthesized compound **C3**

*Compound* **C4**

Crystallized from the hot mixed solvent of ethanol and water (v:v=10:1) to obtain crystals in colorless needles, mp 260-262 ˚C; IR (KBr) cm^-1^: 3417.08, 3218.51, 3100.00, 2946.26, 1703.56, 1668.33, 1613.88, 1585.05, 1521.00, 1485.77, 752.31; ^1^H NMR (400 MHz, DMSO-d^6^) δ=9.72 (s, 1H, CONH), 8.68 (s, 1H, NH on the pyrimidine ring), 7.61 (s, 1H, NH on the pyrimidine ring), 6.89-7.59 (m, 8H, H on the benzene ring), 5.64 (s, 1H, CH), 3.89-3.97 (q, 2H, CH_2_CH_3_), 1.99 (s, 3H, CH_3_ on the pyrimidine ring), 1.23 (t, 3H, CH_2_CH_3_); ESI-MS calcd. For C_20_H_20_ClN_3_O_3_, m/z: 385.1193, found 384.1125 [M-H]^-^.

**Figure S7**. IR spectra of synthesized compound **C4**

**Figure S8**. ^1^HNMR spectra of synthesized compound **C4**

*Compound* **C5**

Crystallized from the hot mixed solvent of ethanol and water (v:v=10:1) to obtain crystals in colorless needles, mp 257-259 ˚C; IR (KBr) cm^-1^: 3480.97, 3384.89, 3247.18, 2952.53, 2930.11, 2827.63, 1697.08, 1661.85, 1636.23, 1584.99, 1517.73, 1488.91, 816.35; ^1^H NMR (400 MHz, DMSO-d^6^) δ=9.72 (s, 1H, CONH), 8.68 (s, 1H, NH on the pyrimidine ring), 7.62 (s, 1H, NH on the pyrimidine ring), 7.20-7.60 (m, 8H, H on the benzene ring), 5.63 (s, 1H, CH), 3.64 (s, 3H, OCH_3_), 2.00 (s, 3H, CH_3_ on the pyrimidine ring); ESI-MS calcd. For C_19_H_18_ClN_3_O_3_, m/z: 371.1037, found 370.0953 [M-H]^-^.

**Figure S9**. IR spectra of synthesized compound **C5**

**Figure S10**. ^1^HNMR spectra of synthesized compound **C5**

*Compound* **C6**

Crystallized from the hot mixed solvent of ethanol and water (v:v=10:1) to obtain crystals in colorless needles, mp 234-236 ˚C; IR (KBr) cm^-1^: 3295.37, 3266.55, 3199.29, 2981.49, 2936.65, 1725.98, 1665.12, 1594.66, 1549.82, 1495.37, 1444.13, 758.72; ^1^H NMR (400MHz, DMSO-d^6^) δ=10.16 (s, 1H, NH on the pyrimidine ring), 7.50 (s, 1H, CONH), 7.48 (s, 1H, NH on the pyrimidine ring), 7.28-6.72 (m, 9H, H on the benzene ring), 4.38 (s, 1H, OH), 4.00-4.06 (q, 1H, CH), 1.68 (s, 3H, CH_3_ on the pyrimidine ring); ESI-MS calcd. For C_18_H_17_N_3_O_3_, m/z: 323.1270, found 322.1195 [M-H]^-^.

**Figure S11**. IR spectra of synthesized compound **C6**

**Figure S12**. ^1^HNMR spectra of synthesized compound **C6**

*Compound* **C7**

Crystallized from the hot mixed solvent of ethanol and water (v:v=10:1) to obtain crystals in colorless needles, mp 289-291 ˚C; IR (KBr) cm^-1^: 3372.24, 3231.32, 3109.61, 2965.48, 2930.25, 1697.15, 1639.50, 1495.37, 1460.14, 752.31; ^1^H NMR (400MHz, DMSO-d^6^) δ=9.00 (s, 1H, NH on the pyrimidine ring), 7.20 (s, 1H, NH on the pyrimidine ring), 7.05-6.94 (m, 4H, H on the benzene ring), 5.42 (s, 1H, CH), 4.03-3.39 (q, 2H, CH_2_CH_3_), 2.23 (s, 3H, CH_3_), 1.36 (t, 3H, CH_2_CH_3_), 1.21 (s, 9H, (CH_3_)_3_); ESI-MS calcd. For C_18_H_24_N_2_O_4_, m/z: 332.1736, found 331.1659 [M-H]^-^.

**Figure S13**. IR spectra of synthesized compound **C7**

**Figure S14**. ^1^HNMR spectra of synthesized compound **C7**

*Compound* **C8**

Crystallized from the hot mixed solvent of ethanol and water (v:v=10:1) to obtain crystals in colorless needles, mp 245-247˚C; IR (KBr) cm^-1^: 3474.73, 3436.30, 3247.33, 2923.84, 2846.98, 1671.53, 1642.70, 1514.59, 1376.87, 736.30; ^1^H NMR (400MHz, DMSO-d^6^) δ=9.75 (s, 1H, NH on the pyrimidine ring), 8.17 (s, 1H, CONH), 7.86 (s, 1H, NH on the pyrimidine ring), 7.84-7.12 (m, 8H, H on the benzene ring), 5.71 (s, 1H, CH), 2.51 (s, 3H, CH_3_ on the pyrimidine ring), 1.96 (s, 3H, CH_3_ on the benzene ring); ESI-MS calcd. For C_19_H_18_N_4_O_4_, m/z: 366.1328, found 365.1252 [M-H]^-^.

**Figure S15**. IR spectra of synthesized compound **C8**

**Figure S16**. ^1^HNMR spectra of synthesized compound **C8**

*Compound* **C9**

Crystallized from the hot mixed solvent of ethanol and water (v:v=10:1) to obtain pale yellow crystals, mp 253-257˚C; IR (KBr) cm^-1^: 3407.47, 3266.55, 3173.67, 2975.09, 2930.25, 1665.12, 1620.28, 1569.04, 1511.39, 1495.37, 758.72; ^1^H NMR (400MHz, DMSO-d^6^) δ=9.93 (s, 1H, CONH), 9.89 (s, 1H, NH on the pyrimidine ring), 9.08 (s, 1H, NH on the pyrimidine ring), 6.91-7.63 (m, 8H, H on the benzene ring), 5.61 (s, 1H, CH), 3.85-3.94 (q, 2H, CH_2_CH_3_), 1.96 (s, 1H, CH_3_ on the pyrimidine ring), 1.17 (t, 3H, CH_2_CH_3_); ESI-MS calcd. For C_20_H_20_ClN_3_O_2_S, m/z: 401.0965, found 400.0890 [M-H]^-^.

**Figure S17**. IR spectra of synthesized compound **C9**

**Figure S18**. ^1^HNMR spectra of synthesized compound **C9**

*Compound* **C10**

Crystallized from the hot mixed solvent of ethanol and water (v:v=10:1) to obtain pale yellow crystals, mp 245-247 ˚C; IR (KBr) cm^-1^: 3417.08, 3269.75, 2930.25, 2879.00, 1668.33, 1626.69, 1565.84, 1488.97, 1444.13, 755.52; ^1^HNMR(400 MHz, DMSO-d^6^) δ=9.86 (s, 1H, NH on the pyrimidine ring), 9.09 (s, 1H, NH on the pyrimidine ring), 8.92 (s, 1H, CONH), 6.92-7.20 (m, 8H, H on the benzene ring), 5.64 (s, 1H, CH), 3.96-4.01 (q, 2H, CH_2_CH_3_), 2.07 (s, 3H, CH_3_ on the pyrimidine ring), 1.91 (s, 3H, CH_3_ on the benzene ring), 1.28 (t, 3H, CH_2_CH_3_); ESI-MS calcd. For C_21_H_23_N_3_O_2_S, m/z: 381.1511, found 380.1441 [M-H]^-^.

**Figure S19**. IR spectra of synthesized compound **C10**

**Figure S20**. ^1^HNMR spectra of synthesized compound **C10**

*Compound* **C11**

Crystallized from the hot mixed solvent of ethanol and water (v:v=10:1) to obtain pale yellow crystals, mp 237-239 ˚C; IR (KBr)cm^-1^: 3413.88, 3282.56, 3180.07, 2936.65, 2885.41, 1626.69, 1661.92, 1572.21, 1488.97, 1437.72, 755.52; ^1^H NMR (400MHz, DMSO-d^6^) δ=9.90 (s, 1H, NH on the pyrimidine ring), 9.73 (s, 1H, NH on the pyrimidine ring), 9.04 (s, 1H, CONH), 6.91-7.27 (m, 9H, H on the benzene ring), 5.63 (s, 1H, CH), 3.86-3.95 (q, 2H, CH_2_CH_3_), 1.97 (s, 3H, CH_3_ on the pyrimidine ring), 1.18 (t, 3H, CH_2_CH_3_). ESI-MS calcd.For C_20_H_21_N_3_O_2_S, m/z: 367.1354, found 366.1279 [M-H]^-^.

**Figure S21**. IR spectra of synthesized compound **C11**

**Figure S22**. ^1^HNMR spectra of synthesized compound **C11**

*Compound* **C12**

Crystallized from the hot mixed solvent of ethanol and water (v:v=10:1) to obtain pale yellow crystals, mp 247-249 ˚C; IR (KBr) cm^-1^: 3298.58, 3183.27, 2994.31, 2927.05, 1684.34, 1629.89, 1594.66, 1514.59, 1437.72, 755.52; ^1^H NMR (400MHz, DMSO-d^6^) δ=9.96 (s, 1H, NH on the pyrimidine ring), 9.72 (s, 1H, NH on the pyrimidine ring), 9.39 (s, 1H, CONH), 6.80-7.56 (m, 8H, H on the benzene ring), 5.36 (s, 1H, CH), 3.71 (s, 3H, OCH_3_), 3.67 (s, 3H, OCH_3_), 2.06 (s, 3H, CH_3_ on the pyrimidine ring). ESI-MS calcd. For C_20_H_21_N_3_O_3_S, m/z:383.1304, found 382.1228 [M-H]^-^.

**Figure S23**. IR spectra of synthesized compound **C12**

**Figure S24**. ^1^HNMR spectra of synthesized compound **C12**

*Compound* **C13**

Crystallized from the hot mixed solvent of ethanol and water (v:v=10:1) to obtain pale yellow crystals, mp 252-254 ˚C; IR (KBr) cm^-1^: 3304.98, 3183.27, 2978.29, 2933.45, 2837.37, 1681.14, 1639.50, 1591.46, 1575.44, 1511.39, 819.57; ^1^H NMR (400MHz, DMSO-d^6^) δ=9.99 (s, 1H, NH on the pyrimidine ring), 9.84 (s, 1H, CONH), 9.42 (s, 1H, NH on the pyrimidine ring), 6.76-7.60 (m, 7H, H on the benzene ring), 5.36 (s, 1H, CH), 3.71 (s, 3H, OCH_3_), 3.67 (s, 3H, OCH_3_), 2.06 (s, 3H, CH_3_ on the pyrimidine ring); ESI-MS calcd. For C_20_H_20_ClN_3_O_3_S, m/z: 417.0914, found 416.0837 [M-H]^-^.

**Figure S25**. IR spectra of synthesized compound **C13**

**Figure S26**. ^1^HNMR spectra of synthesized compound **C13**

*Compound* **C14**

Crystallized from the hot mixed solvent of ethanol and water (v:v=10:1) to obtain pale yellow crystals, mp 263-265˚C; IR (KBr) cm^-1^: 3253.74, 3029.54, 2917.44, 2853.38, 1671.53, 1642.70, 1585.05, 1517.79, 1456.94, 736.30; ^1^H NMR (400MHz, DMSO-d^6^) δ= 9.72 (s, 1H, NH on the pyrimidine ring), 8.25 (s, 1H, NH on the pyrimidine ring), 8.15 (s, 1H, CONH), 7.07-7.72 (m, 8H, H on the benzene ring), 5.48 (s, 1H, CH), 2.48 (s, 1H, CH_3_ on the pyrimidine ring), 1.94 (s, 3H, CH_3_ on the benzene ring); ESI-MS calcd. For C_19_H_18_N_4_O_3_S, m/z: 382.1100, found 381.1023 [M-H]^-^.

**Figure S27**. IR spectra of synthesized compound **C14**

**Figure S28**. ^1^HNMR spectra of synthesized compound **C14**

*Compound* **D1**

Crystallized from the hot mixed solvent of ethanol and DMF (v:v=10:1) to obtain pale yellow crystals, mp 231-233˚C;IR (KBr) cm^-1^: 3413.72, 3237.57, 2981.36, 2952.53, 1716.30, 1668.26, 1610.61, 1453.68, 752.36; ^1^H NMR (400MHz, DMSO-d^6^) δ=11.68 (s, 1H, NH on the pyrrole ring), 8.86 (s, 1H, NH on the pyrimidine ring), 8.55 (s, 1H, NH on the pyrimidine ring), 7.94 (s, 1H, CONH), 6.97-7.01 (m, 4H, H on the benzene ring), 6.10 (s, 1H, CH), 4.21-4.24 (q, 2H, CH_2_CH_3_), 3.78 (s, 3H, COOCH_3_), 3.76 (s, 3H, OCH_3_), 2.47 (s, 3H, CH_3_ on the pyrrole ring), 2.14 (s, 3H, CH_3_ on the pyrimidine ring), 1.28 (t, 3H, CH_2_CH_3_); ESI-MS calcd. For C_23_H_26_N_4_O_7_, m/z: 470.1801, found 469.1725 [M-H]^-^.

**Figure S29**. IR spectra of synthesized compound **D1**

**Figure S30**. ^1^HNMR spectra of synthesized compound **D1**

*Compound* **D2**

Crystallized from the hot mixed solvent of ethanol and DMF (v:v=10:1) to obtain pale yellow crystals, mp 228-230 ˚C; IR (KBr) cm^-1^: 3410.52, 3243.98, 2978.15, 2949.33, 1716.30, 1681.07, 1661.85, 1610.61, 1514.53, 1460.09, 752,36; ^1^H NMR (400MHz, DMSO-d^6^) δ=11.68 (s, 1H, NH on the pyrrole ring), 8.86 (s, 1H, NH on the pyrimidine ring), 8.55 (s, 1H, NH on the pyrimidine ring), 7.95 (s, 1H, CONH), 6.97-7.01 (m, 4H, H on the benzene ring), 6.10 (s, 1H, CH), 4.21-4.24 (q, 2H, CH_2_CH_3_), 3.76 (s, 3H, CH_3_ on the pyrrole ring), 3.76 (s, 3H, COOCH_3_), 2.50 (s, 3H, CH_3_), 2.47 (s, 3H, CH_3_ on the pyrimidine ring), 2.14 (s, 3H, CH_3_ on the benzene ring), 1.28 (t, 3H, CH_2_CH_3_); ESI-MS calcd. For C_23_H_26_N_4_O_6_, m/z: 454.1852, found 453.1783[M-H]^-^.

**Figure S31**. IR spectra of synthesized compound **D2**

**Figure S32**. ^1^HNMR spectra of synthesized compound **D2**

*Compound* **D3**

Crystallized from the hot mixed solvent of ethanol and DMF (v:v=10:1) to obtain pale yellow crystals, mp 233-235 ˚C; IR (KBr) cm^-1^: 3404.11, 3247.18, 2974.95, 2949.33, 1706.69, 1668.26, 1610.51, 1524.14, 1456.38, 751.35; ^1^H NMR (400MHz, DMSO-d^6^) δ=11.67 (s, 1H, NH on the pyrrole ring), 8.85 (s, 1H, NH on the pyrimidine ring), 8.57 (s, 1H, NH on the pyrimidine ring), 7.93 (m, 1H, CONH), 6.99-7.03 (m, 4H, H on the benzene ring), 6.05 (s, 1H, CH), 4.26 (q, 2H, CH_2_CH_3_), 3.76 (s, 3H, COOCH_3_), 3.75 (s, 3H, OCH_3_), 2.46 (s, 3H, CH_3_ on the pyrrole ring), 2.13 (s, 3H, CH_3_ on the pyrimidine ring), 1.27 (t, 3H, CH_2_CH_3_); ESI-MS calcd. For C_23_H_26_N_4_O_7_, m/z: 470.1801, found 469.1726 [M-H]^-^.

**Figure S33**. IR spectra of synthesized compound **D3**

**Figure S34**. ^1^HNMR spectra of synthesized compound **D3**

*Compound* **D4**

Crystallized from the hot mixed solvent of ethanol and DMF (v:v=10:1) to obtain pale yellow crystals, mp 234-236 ˚C; IR (KBr) cm^-1^: 3397.71, 3243.98, 2968.55, 2949.33, 1709.89, 1671.46, 1610.61, 1520.91, 1450.48, 750.48; ^1^H NMR (400MHz, DMSO-d^6^) δ=11.50 (s, 1H, NH on the pyrrole ring), 8.81 (s, 1H, CONH), 8.75 (s, 1H, NH on the pyrimidine ring), 7.22 (d, J=7.6 Hz, 1H, NH on the pyrimidine ring), 7.03-7.13 (m, 4H, H on the benzene ring), 6.03 (s, 1H, CH), 4.24-4.30 (q, 2H, CH_2_CH_3_), 3.72 (s, 3H, COOCH_3_), 2.44 (s, 3H, CH_3_ on the pyrrole ring), 2.08 (s, 3H, CH_3_ on the pyrimidine ring), 1.94 (s, 3H, CH_3_ on the benzene ring),1.28 (t, 3H, CH_2_CH_3_); ESI-MS calcd. For C_23_H_26_N_4_O_6_, m/z: 454.1852, found 453.1774 [M-H]^-^.

**Figure S35**. IR spectra of synthesized compound **D4**

**Figure S36**. ^1^HNMR spectra of synthesized compound **D4**

*Compound* **D5**

Crystallized from the hot mixed solvent of ethanol and DMF (v:v=10:1) to obtain pale yellow crystals, mp 234-236˚C;IR(KBr)cm^-1^3: 3413.72, 3247.18, 2971.75, 2949.33, 1706.69, 1668.26, 1620.22, 1591.40, 1514.53, 1450.48, 751.49; ^1^H NMR (400MHz, DMSO-d^6^) δ=11.47 (s, 1H, NH on the pyrrole ring), 8.81 (s, 1H, CONH), 8.79 (s, 1H, NH on the pyrimidine ring), 7.25 (d, J=7.7 Hz, 1H, NH on the pyrimidine ring), 6.99-7.13 (m, 4H, H on the benzene ring), 6.08 (s, 1H, CH), 4.19-4.28 (q, 4H, 2CH_2_CH_3_), 2.45 (s, 3H, CH_3_ on the pyrrole ring), 2.08 (s, 3H, CH_3_ on the pyrimidine ring), 1.98 (s, 3H, CH_3_ on the benzene ring), 1.27 (t, 3H, CH_2_CH_3_), 1.28 (t, 3H, CH_2_CH_3_); ESI-MS calcd. For C_24_H_28_N_4_O_6_, m/z: 468.2009, found 467.1930[M-H]^-^.

**Figure S37**. IR spectra of synthesized compound **D5**

**Figure S38**. ^1^HNMR spectra of synthesized compound **D5**

*Compound* **D6**

Crystallized from the hot mixed solvent of ethanol and DMF (v:v=10:1) to obtain pale yellow crystals, mp 224-226˚C; IR (KBr) cm^-1^: 3410.39, 3247.01, 2952.07, 1719.00, 1683.76, 1661.34, 1597.27, 1526.80, 1446.71, 750.39; ^1^H NMR (400MHz, DMSO-d^6^) δ=11.56 (s, 1H, NH on the pyrrole ring), 8.80 (s, 1H, CONH), 8.76 (s, 1H, NH on the pyrimidine ring), 7.22 (d, J=7.2 Hz, 1H, NH on the pyrimidine ring), 7.03-7.13 (m, 4H, H on the benzene ring), 6.04 (s, 1H, CH), 3.79 (s, 3H, COOCH_3_), 3.72 (s, 3H, COOCH_3_), 2.44 (s, 3H, CH_3_ on the pyrrole ring), 2.08 (s, 3H, CH_3_ on the pyrimidine ring), 1.94(s, 3H, CH_3_ on the benzene ring); ESI-MS calcd. For C_22_H_24_N_4_O_6_, m/z: 440.1696, found 439.1619[M-H]^-^.

**Figure S39**. IR spectra of synthesized compound **D6**

**Figure S40**. ^1^HNMR spectra of synthesized compound **D6**

*Compound* **D7**

Crystallized from the hot mixed solvent of ethanol and DMF (v:v=10:1) to obtain pale yellow crystals, mp 240-242 ˚C; IR (KBr) cm^-1^: 3420.12, 3279.21, 2978.15, 2986.52, 1709.89, 1668.26, 1620.22, 1524.14, 1453.68, 776.03; ^1^H NMR (400MHz, DMSO-d^6^) δ=11.32 (s, 1H, NH on the pyrrole ring), 9.60 (s, 1H, CONH), 8.83 (s, 1H, NH on the pyrimidine ring), 7.51-7.30 (m, 4H, H on the benzene ring), 7.09 (s, 1H, NH on the pyrimidine ring), 6.05 (s, 1H, CH), 4.17-4.27 (q, 4H, 2CH_2_CH_3_), 2.42 (s, 3H, CH_3_ on the pyrrole ring), 2.03 (s, 3H, CH_3_ on the pyrimidine ring), 1.28 (t, 3H, CH_2_CH_3_), 1.27 (t, 3H, CH_2_CH_3_); ESI-MS calcd. For C_23_H_25_ClN_4_O_6_, m/z: 488.1463, found 487.1387 [M-H]^-^.

**Figure S41**. IR spectra of synthesized compound **D7**

**Figure S42**. ^1^HNMR spectra of synthesized compound **D7**

*Compound* **D8**

Crystallized from the hot mixed solvent of ethanol and DMF (v:v=10:1) to obtain pale yellow crystals, mp 236-238 ˚C;IR (KBr) cm^-1^: 3407.31, 3253.58, 2978.15, 2949.33, 1713.10, 1687.48, 1665.06, 1607.41, 1524.14, 1492.11, 1444.07, 777.92; ^1^H NMR (400MHz, DMSO-d^6^) δ=11.37 (s, 1H, NH on the pyrrole ring), 9.57 (s, 1H, CONH), 8.81 (s, 1H, NH on the pyrimidine ring), 7.30-7.52 (m, 4H, H on the benzene ring), 7.14 (s, 1H, NH on the pyrimidine ring), 6.02 (s, 1H, CH), 4.23-4,29 (q, 2H, CH_2_CH_3_), 3.70 (s, 3H, COOCH_3_), 2.42 (s, 3H, CH_3_ on the pyrrole ring), 2.02 (s, 3H, CH_3_ on the pyrimidine ring), 1.28 (t, 3H, CH_2_CH_3_); ESI-MS calcd. For C_22_H_23_ClN_4_O_6_, m/z: 474.1306, found 473.1227[M-H]^-^.

**Figure S43**. IR spectra of synthesized compound **D8**

**Figure S44**. ^1^HNMR spectra of synthesized compound **D8**

*Compound* **D9**

Crystallized from the hot mixed solvent of ethanol and DMF (v:v=10:1) to obtain pale yellow crystals, mp 229-231˚C; IR (KBr) cm^-1^: 3404.27, 3250.58, 2949.47, 2930.25, 1709.96, 1687.54, 1661.92, 1613.88, 1588.26, 1521.00, 1488.97, 1450.53, 771.53; ^1^H NMR (400MHz, DMSO-d^6^) δ= 11.40 (s, 1H, NH on the pyrrole ring), 9.48 (s, 1H, CONH), 8.80 (s, 1H, NH on the pyrimidine ring), 7.27-7.49 (m, 5H, H on the benzene ring), 7.25 (t, J=7.4 Hz, 1H, NH on the pyrimidine ring), 6.05 (s, 1H, CH), 3.80 (s, 3H, OCH_3_), 3.73 (s, 3H, OCH_3_), 2.43 (s, 3H, CH_3_ on the pyrrole ring), 2.04 (s, 3H, CH_3_ on the pyrimidine ring); ESI-MS calcd. For C_21_H_22_N_4_O_6_, m/z: 426.1539, found 425.1458[M-H]^-^.

**Figure S45**. IR spectra of synthesized compound **D9**

**Figure S46**. ^1^HNMR spectra of synthesized compound **D9**

*Compound* **D10**

Crystallized from the hot mixed solvent of ethanol and DMF (v:v=10:1) to obtain pale yellow crystals, mp 233-235˚C; IR (KBr) cm^-1^: 3413.38, 3256.94, 2978.29, 2955.87, 1716.37, 1668.33, 1613.88, 1591.46, 1530.60, 1437.72, 749.11; ^1^H NMR (400 MHz, DMSO-d^6^) δ=11.34 (s, 1H, NH on the pyrrole ring), 9.48 (s, 1H, CONH), 8.80 (s, 1H, NH on the pyrimidine ring), 7.47 (s,1H, NH on the pyrimidine ring), 7.04-7.46 (m, 5H, H on the benzene ring), 6.06 (s, 1H, CH), 4.16-4.19 (q, 2H, CH_2_CH_3_), 3.79 (s, 3H, COOCH_3_), 2.43 (s, 3H, CH_3_ on the pyrrole ring), 2.04 (s, 3H, CH_3_ on the pyrimidine ring), 1.26 (t, 3H, CH_2_CH_3_); ESI-MS calcd. For C_22_H_24_N_4_O_6_, m/z: 440.1696, found 439.1628[M-H]^-^.

**Figure S47**. IR spectra of synthesized compound **D10**

**Figure S48**. ^1^HNMR spectra of synthesized compound **D10**

*Compound* **D11**

Crystallized from the hot mixed solvent of ethanol and DMF (v:v=10:1) to obtain pale yellow crystals, mp 247-249˚C; IR (KBr) cm^-1^: 3420.00, 3243.81, 2974.73, 2936.29, 1709.39, 1670.95, 1606.88, 1526.80, 1443.51, 751.58; ^1^H NMR (400 MHz, DMSO-d^6^) δ=11.30 (s, 1H, NH on the pyrrole ring), 9.49 (s, 1H, CONH), 8.81 (s, 1H, NH on the pyrimidine ring), 7.48 (s, 1H, NH on the pyrimidine ring), 7.04-7.46 (m, 5H, H on the benzene ring), 6.05 (s, 1H, CH), 4.18-4.27 (q, 4H, 2CH_2_CH_3_), 2.43 (s, 3H, CH_3_ on the pyrrole ring), 2.03 (s, 3H, CH_3_ on the pyrimidine ring), 1.27 (t, 3H, CH_2_CH_3_), 1.28 (t, 3H, CH_2_CH_3_); ESI-MS calcd. For C_23_H_26_N_4_O_6_, m/z: 454.1852, found 453.1767 [M-H]^-^.

**Figure S49**. IR spectra of synthesized compound **D11**

**Figure S50**. ^1^HNMR spectra of synthesized compound **D11**

*Compound* **D12**

Crystallized from the hot mixed solvent of ethanol and DMF (v:v=10:1) to obtain pale yellow crystals, m.p. 237-239 ˚C; IR (KBr) cm^-1^: 3388.10, 3221.56, 2974.95, 1709.89, 1671.46, 1572.18, 1434.46, 771.51; ^1^H NMR (400 MHz, DMSO-d^6^) δ=1.28 (t, 6H, 2CH_2_CH_3_), 2.24 (s, 3H, CH_3_ on the pyrimidine ring), 2.45(s, 3H, CH_3_ on the pyrrole ring), 3.43 (s, 3H, COOCH_3_), 4.24-4.27 (q, 4H, 2CH_2_CH_3_), 5.98 (s, 1H, CH), 9.05 (s, 1H, NH on the pyrimidine ring), 10.25 (s, 1H, NH on the pyrimidine ring), 11.87 (s, 1H, NH on the pyrrole ring); ESI-MS calcd. For C_18_H_23_N_3_O_6_S, m/z: 409.1308, found 408.1230[M-H]^-^.

**Figure S51**. IR spectra of synthesized compound **D12**

**Figure S52**. ^1^HNMR spectra of synthesized compound **D12**

*Compound* **D13**

Crystallized from the hot mixed solvent of ethanol and DMF (v:v=10:1) to obtain pale yellow crystals, m.p.255-256˚C; IR (KBr) cm^-1^: 3372.24, 3285.77, 2987.90, 1713.17, 1687.54, 1655.52, 1575.44, 1460.14, 771.50; ^1^H NMR (400 MHz, DMSO-d^6^) δ=0.92 (t, 3H, CH_2_CH_3_), 2.23 (s, 3H, CH_3_ on the pyrimidine ring), 2.45 (s, 3H, CH_3_ on the pyrrole ring), 3.71 (s, 3H, COOCH_3_), 3.79 (s, 3H, COOCH_3_), 3.80 (q, 2H, CH_2_CH_3_), 5.97 (s, 1H, CH), 9.18 (s, 1H, NH on the pyrimidine ring), 10.19 (s, 1H, NH on the pyrimidine ring), 11.98 (s, 1H, NH on the pyrrole ring); ESI-MS calcd. For C_17_H_21_N_3_O_6_S, m/z: 395.1151, found 394.1071[M-H]^-^.

**Figure S53**. IR spectra of synthesized compound **D13**

**Figure S54**. ^1^HNMR spectra of synthesized compound **D13**

*Compound* **D14**

Crystallized from the hot mixed solvent of ethanol and DMF (v:v=10:1) to obtain pale yellow crystals, m.p.228-230˚C; IR (KBr) cm^-1^: 3413.72, 3298.42, 2952.53, 1725.91, 1687.48, 1610.61, 1559.37, 1450.48, 701.13; ^1^H NMR (400 MHz, DMSO-d^6^) δ=1.60 (s, 3H, CH_3_ on the pyrimidine ring), 2.38 (s, 3H, CH_3_ on the pyrrole ring), 3.73 (s, 3H, COOCH_3_), 3.77 (s, 3H, COOCH_3_), 6.16 (s, 1H, CH), 7.49-7.55 (m, 5H, H on the benzene ring), 9.20 (s, 1H, NH on the pyrimidine ring)，10.13 (s, 1H, NH on the pyrimidine ring), 12.14 (s, 1H, NH on the pyrrole ring); ESI-MS calcd. For C_21_H_21_N_3_O_5_S, m/z: 427.1202, found 426.1121[M-H]^-^.

**Figure S55**. IR spectra of synthesized compound **D14**

**Figure S56**. ^1^HNMR spectra of synthesized compound **D14**

*Compound* **D15**

Crystallized from the hot mixed solvent of ethanol and DMF (v:v=10:1) to obtain pale yellow crystals, m.p.212-214˚C; IR (KBr) cm^-1^: 3276.00, 2981.36, 2952.53, 1716.30, 1684.27, 1572.18, 1444.07, 701.01; ^1^H NMR (400 MHz, DMSO-d^6^) δ=1.30 (t, 3H, CH_2_CH_3_), 1.60 (s, 3H, CH_3_ on the pyrimidine ring), 2.39 (s, 3H, CH_3_ on the pyrrole ring), 3.78 (s, 3H, COOCH_3_), 4.17-4,24 (q, 2H, CH_2_CH_3_), 6.20 (s, 1H, CH), 7.39-7.59 (m, 5H, H on the benzene ring), 9.18(s, 1H, NH on the pyrimidine ring), 10.12(s, 1H, NH on the pyrimidine ring), 12.13(s, 1H, NH on the pyrrole ring); ESI-MS calcd. For C_22_H_23_N_3_O_5_S, m/z: 441.1358, found 440.1278[M-H]^-^.

**Figure S57**. IR spectra of synthesized compound **D15**

**Figure S58**. ^1^HNMR spectra of synthesized compound **D15**

*Compound* **D16**

Crystallized from the hot mixed solvent of ethanol and DMF (v:v=10:1) to obtain pale yellow crystals, mp 231-233˚C; IR (KBr) cm^-1^: 3410.39, 3265.45, 2977.93, 2955.51, 1719.00, 1702.99, 1670.95, 1619.70, 1558.83, 1536.41, 1465.94, 1440.31, 751.58; ^1^H NMR (400 MHz, DMSO-d^6^) δ=11.59 (s, 1H, NH on the pyrrole ring), 10.01 (s, 1H,NH on the pyrimidine ring), 9.54 (s, 1H, NH on the pyrimidine ring), 8.87 (s, 1H, CONH), 7.02-7.47 (m, 5H, H on the benzene ring), 6.11 (s, 1H, CH), 4.19-4.21 (q, 2H, CH_2_CH_3_), 3.80 (s, 3H, COOCH_3_), 2.43 (s, 3H, CH_3_ on the pyrrole ring), 2.04 (s, 3H, CH_3_ on the pyrimidine ring), 1.26 (t, 3H, CH_2_CH_3_); ESI-MS calcd. For C_22_H_24_N_4_O_5_S, m/z: 456.1467, found 455.1386[M-H]^-^.

**Figure S59**. IR spectra of synthesized compound **D16**

**Figure S60**. ^1^HNMR spectra of synthesized compound **D16**

**Expression and purification of Eg5368H:**

A sequence of human Eg5 corresponding to amino acids 1 to 368 was amplified with polymerase chain reaction (PCR) by NOVOPRO BIOSCIENCE INC. It was cloned into pET22b introducing a C-terminal His-tag. The protein was expressed in *Escherichia coli* BL21 (DE3) cells for 12 h at 25 ℃ in LB containing 1mM Isopropyl-beta-D-thiogalactopyranoside (IPTG), 50 ug/ml Ampicillin. The harvested cells were lysed with Ultrasonic Processor in Binder-buffer (50 mM Tris-HCl, 350 mM NaCl, 10 mM Imidazole, pH 8.0). The clarified lysate was loaded onto a Ni-IDA -Sepharose CL-6B Affi Gel column (Sigma), washed with Washing-buffer(20 mM Tris-HCl, 350 mM NaCl, 20 mM Imidazole, pH 8.0) untill effluent OD280 value reaches baseline. Then it was eluted with Elution-buffer (20 mM Tris-HCl, 350 mM NaCl , 250 mM Imidazole, pH8.0). The buffer was exchanged to 150 mM NaCl, 20mM Tris pH 8.0 by dialysis. The protein concentration was adjusted to 2 mg/ml (measured by a Bradford assay using bovine serum albumin (BSA) as standard). Eg5368H was stored at -20 ℃.

**Tubulin Preparation:**

Pig brains were cleaned of blood clots with kitchen paper, weighed, and transferred to a Waring blender. Cold (4 ℃) depolymerization buffer (DB) was added at a ratio of 1 liter/kg of brain tissue. The mixture was homogenized twice for approximately 30 s. The homogenates were then centrifuged in a SLA 1500 Sorvall rotor at 29,000g for 60 min at 4 ℃. The supernatants were pooled and supplemented with an equal volume of warm (37 ℃) high-molarity PIPES buffer (HMPB), ATP (1.5 mM final), and GTP (0.5 mM final). An equal volume (1/3 of the final volume) of pre-warmed to 37 ℃ anhydrous glycerol was added to this solution. This mixture was incubated in a 37 ℃ water bath for 1 h. The polymerized tubulin was then centrifuged in a Ti 45 Beckman rotor at 151,000g (44,000 rpm) for 30 min at 37 ℃. The resulting microtubule pellets were resuspended in 100 ml of cold DB and left for 30 min on ice. The depolymerized tubulinwas subsequently centrifuged in a Ti 45 Beckman rotor at 70,000g (30,000 rpm) for 30 min at 4 ℃. The supernatant from this centrifugation step was mixed with an equal volume of the HMPB supplemented with ATP and GTP, followed by the addition of glycerol (1/3 of the final volume) as described above. The mixture was incubated in a 37 ℃ water bath for 30 min, and the polymerized tubulin was pelleted in a Ti 45 Beckman rotor at 151,000g (44,000 rpm) for 30 min at 37 ℃. Following the centrifugation, the microtubule pellets were resuspended in 15 ml of ice-cold BRB80 and then incubated for a further 10 min on ice. After this depolymerization step, the tubulin was centrifuged in a TLA 100.4 Beckman tabletop ultracentrifuge rotor at 104,000g (50,000 rpm) for 30 min at 4 ℃. The tubulin supernatant was collected and stored at -60 ℃.

**Microtubule polymerization in vitro:**

A mixture of 500 µl purified pig brain tubulin , 500 µl BRB80 (80 mM PIPES pH 6.8, 1 mM MgCl2 and 1 mM EGTA), 337 uL glycerol and 1.25 µl 100 mM GTP was incubated at 37 °C for 30 minutes. Polymerized microtubules were then stabilized by addition of 10 µl 10 mM paclitaxel (Sigma) in DMSO and incubated at 37 °C for another 20 min. The suspension was centrifuged at 70000 rpm at 30 °C for 30 minutes in a TLA100 rotor (Beckman). The resulting microtubule pellet was resuspended in 588 µl BRB80 supplemented with 12 ul 1 mM paclitaxel at 37 °C. This microtubule suspension was stored at room temperature. The final concentration was 60 µM polymerized tubulin as determined by Bradford using BSA as a standard.

**Identification of Potential Eg5 motor protein Inhibitors by Screening In vitro:**

Table S1. OD630 values for inhibition of microtubule-stimulated KSP ATPase activity

| **Entry** | **Concentration uM** | **OD630 Value** | | | **Mean** | **SD** |
| --- | --- | --- | --- | --- | --- | --- |
| **C9** | 121.21 | 0.141 | 0.14 | 0.14 | 0.14 | 0.001 |
| **C9** | 90.91 | 0.155 | 0.157 | 0.155 | 0.156 | 0.001 |
| **C9** | 80.81 | 0.16 | 0.155 | 0.156 | 0.157 | 0.003 |
| **C9** | 60.61 | 0.166 | 0.163 | 0.161 | 0.163 | 0.003 |
| **C9** | 30.3 | 0.186 | 0.18 | 0.179 | 0.182 | 0.004 |
| **C9** | 24.24 | 0.221 | 0.223 | 0.218 | 0.221 | 0.003 |
| **Negative** | 0 | 0.28 | 0.283 | 0.279 | 0.281 | 0.002 |
| **Blank** | 0 | 0.113 | 0.112 | 0.111 | 0.112 | 0.001 |
| **D1** | 101.52 | 0.16 | 0.158 | 0.158 | 0.159 | 0.001 |
| **D1** | 76.14 | 0.19 | 0.189 | 0.188 | 0.189 | 0.001 |
| **D1** | 67.68 | 0.194 | 0.197 | 0.194 | 0.195 | 0.002 |
| **D1** | 50.76 | 0.249 | 0.248 | 0.246 | 0.248 | 0.002 |
| **D1** | 25.38 | 0.26 | 0.261 | 0.259 | 0.26 | 0.001 |
| **Negative** | 0 | 0.366 | 0.377 | 0.366 | 0.37 | 0.006 |
| **Blank** | 0 | 0.126 | 0.126 | 0.126 | 0.126 | 0 |
| **D2** | 80.81 | 0.137 | 0.139 | 0.136 | 0.137 | 0.002 |
| **D2** | 60.61 | 0.223 | 0.216 | 0.217 | 0.219 | 0.004 |
| **D2** | 53.87 | 0.266 | 0.255 | 0.26 | 0.26 | 0.006 |
| **D2** | 40.41 | 0.296 | 0.272 | 0.269 | 0.279 | 0.015 |
| **D2** | 20.2 | 0.338 | 0.32 | 0.308 | 0.322 | 0.015 |
| **Negative** | 0 | 0.28 | 0.284 | 0.285 | 0.283 | 0.003 |
| **Blank** | 0 | 0.106 | 0.106 | 0.105 | 0.106 | 0.001 |
| **D3** | 121.21 | 0.116 | 0.114 | 0.115 | 0.115 | 0.001 |
| **D3** | 90.91 | 0.123 | 0.122 | 0.121 | 0.122 | 0.001 |
| **D3** | 60.61 | 0.15 | 0.146 | 0.141 | 0.146 | 0.005 |
| **D3** | 30.3 | 0.181 | 0.183 | 0.182 | 0.182 | 0.001 |
| **D3** | 24.24 | 0.22 | 0.218 | 0.217 | 0.218 | 0.002 |
| **Negative** | 0 | 0.199 | 0.206 | 0.204 | 0.203 | 0.004 |
| **Blank** | 0 | 0.115 | 0.116 | 0.115 | 0.115 | 0.001 |
| **D4** | 121.21 | 0.134 | 0.134 | 0.134 | 0.134 | 0 |
| **D4** | 90.91 | 0.151 | 0.146 | 0.147 | 0.148 | 0.003 |
| **D4** | 60.61 | 0.159 | 0.159 | 0.157 | 0.158 | 0.001 |
| **D4** | 30.3 | 0.174 | 0.176 | 0.171 | 0.174 | 0.003 |
| **D4** | 24.24 | 0.199 | 0.197 | 0.198 | 0.198 | 0.001 |
| **Negative** | 0 | 0.199 | 0.206 | 0.204 | 0.203 | 0.004 |
| **Blank** | 0 | 0.115 | 0.116 | 0.115 | 0.115 | 0.001 |
| **D7** | 121.21 | 0.145 | 0.147 | 0.145 | 0.146 | 0.001 |
| **D7** | 90.91 | 0.157 | 0.159 | 0.158 | 0.158 | 0.001 |
| **D7** | 80.81 | 0.163 | 0.164 | 0.164 | 0.164 | 0.001 |
| **D7** | 60.61 | 0.195 | 0.198 | 0.197 | 0.197 | 0.002 |
| **D7** | 30.3 | 0.23 | 0.232 | 0.231 | 0.231 | 0.001 |
| **Negative** | 0 | 0.265 | 0.27 | 0.266 | 0.267 | 0.003 |
| **Blank** | 0 | 0.116 | 0.118 | 0.118 | 0.117 | 0.001 |
| **D10** | 121.21 | 0.138 | 0.134 | 0.127 | 0.133 | 0.006 |
| **D10** | 107.74 | 0.139 | 0.14 | 0.139 | 0.139 | 0.001 |
| **D10** | 90.91 | 0.191 | 0.192 | 0.189 | 0.191 | 0.002 |
| **D10** | 80.81 | 0.199 | 0.198 | 0.193 | 0.197 | 0.003 |
| **D10** | 60.61 | 0.226 | 0.228 | 0.222 | 0.225 | 0.003 |
| **Negative** | 0 | 0.328 | 0.337 | 0.336 | 0.334 | 0.005 |
| **Blank** | 0 | 0.121 | 0.122 | 0.122 | 0.122 | 0.001 |
| **D12** | 121.21 | 0.126 | 0.122 | 0.122 | 0.123 | 0.002 |
| **D12** | 90.91 | 0.129 | 0.129 | 0.13 | 0.129 | 0.001 |
| **D12** | 80.81 | 0.14 | 0.141 | 0.142 | 0.141 | 0.001 |
| **D12** | 60.61 | 0.189 | 0.192 | 0.191 | 0.191 | 0.002 |
| **D12** | 30.3 | 0.239 | 0.243 | 0.246 | 0.243 | 0.004 |
| **Negative** | 0 | 0.265 | 0.27 | 0.266 | 0.267 | 0.003 |
| **Blank** | 0 | 0.116 | 0.118 | 0.118 | 0.117 | 0.001 |
| **D14** | 121.21 | 0.14 | 0.141 | 0.14 | 0.14 | 0.001 |
| **D14** | 90.91 | 0.141 | 0.137 | 0.136 | 0.138 | 0.003 |
| **D14** | 80.81 | 0.158 | 0.148 | 0.148 | 0.151 | 0.006 |
| **D14** | 60.61 | 0.191 | 0.185 | 0.185 | 0.187 | 0.003 |
| **D14** | 30.3 | 0.254 | 0.248 | 0.248 | 0.25 | 0.003 |
| **Negative** | 0 | 0.214 | 0.213 | 0.211 | 0.213 | 0.002 |
| **Blank** | 0 | 0.129 | 0.128 | 0.127 | 0.128 | 0.001 |
| **Monastrol** | 101.52 | 0.175 | 0.178 | 0.177 | 0.177 | 0.002 |
| **Monastrol** | 76.14 | 0.188 | 0.188 | 0.187 | 0.188 | 0.001 |
| **Monastrol** | 67.68 | 0.192 | 0.189 | 0.188 | 0.190 | 0.002 |
| **Monastrol** | 50.76 | 0.195 | 0.192 | 0.192 | 0.193 | 0.002 |
| **Monastrol** | 25.38 | 0.216 | 0.214 | 0.212 | 0.214 | 0.002 |
| **Negative** | 0 | 0.274 | 0.28 | 0.28 | 0.278 | 0.003 |
| **Blank** | 0 | 0.119 | 0.117 | 0.117 | 0.118 | 0.001 |

Table S2. OD630 values for Lineweavere—Burk plot of inhibition of Eg5 by **C9**

| **Entry** | **C9 Concentration uM** | **ATP Concentration mM** | **OD630 Value** | | | **Mean** | **SD** |
| --- | --- | --- | --- | --- | --- | --- | --- |
| **C9** | 60.5 | 0.417 | 0.198 | 0.199 | 0.198 | 0.198 | 0.001 |
| **C9** | 30.25 | 0.417 | 0.231 | 0.232 | 0.23 | 0.231 | 0.001 |
| **C9** | 15.13 | 0.417 | 0.263 | 0.27 | 0.271 | 0.268 | 0.004 |
| **Negative** | 0 | 0.417 | 0.365 | 0.363 | 0.364 | 0.364 | 0.001 |
| **Blank** | 0 | 0.417 | 0.116 | 0.113 | 0.113 | 0.114 | 0.002 |
| **C9** | 60.5 | 0.313 | 0.171 | 0.17 | 0.171 | 0.171 | 0.001 |
| **C9** | 30.25 | 0.313 | 0.197 | 0.196 | 0.197 | 0.197 | 0.001 |
| **C9** | 15.13 | 0.313 | 0.226 | 0.224 | 0.228 | 0.226 | 0.002 |
| **Negative** | 0 | 0.313 | 0.332 | 0.33 | 0.336 | 0.333 | 0.003 |
| **Blank** | 0 | 0.313 | 0.102 | 0.1 | 0.101 | 0.101 | 0.001 |
| **C9** | 60.5 | 0.209 | 0.156 | 0.156 | 0.157 | 0.156 | 0.001 |
| **C9** | 30.25 | 0.209 | 0.173 | 0.174 | 0.174 | 0.174 | 0.001 |
| **C9** | 15.13 | 0.209 | 0.2 | 0.197 | 0.199 | 0.199 | 0.002 |
| **Negative** | 0 | 0.209 | 0.298 | 0.302 | 0.303 | 0.301 | 0.003 |
| **Blank** | 0 | 0.209 | 0.092 | 0.091 | 0.092 | 0.092 | 0.001 |
| **C9** | 60.5 | 0.104 | 0.136 | 0.138 | 0.139 | 0.138 | 0.002 |
| **C9** | 30.25 | 0.104 | 0.155 | 0.155 | 0.156 | 0.155 | 0.001 |
| **C9** | 15.13 | 0.104 | 0.172 | 0.179 | 0.179 | 0.177 | 0.004 |
| **Negative** | 0 | 0.104 | 0.261 | 0.259 | 0.264 | 0.261 | 0.003 |
| **Blank** | 0 | 0.104 | 0.095 | 0.095 | 0.094 | 0.095 | 0.001 |
| **C9** | 60.5 | 0.052 | 0.112 | 0.114 | 0.117 | 0.114 | 0.003 |
| **C9** | 30.25 | 0.052 | 0.127 | 0.126 | 0.125 | 0.126 | 0.001 |
| **C9** | 15.13 | 0.052 | 0.14 | 0.142 | 0.143 | 0.142 | 0.002 |
| **Negative** | 0 | 0.052 | 0.218 | 0.221 | 0.221 | 0.22 | 0.002 |
| **Blank** | 0 | 0.052 | 0.087 | 0.085 | 0.085 | 0.086 | 0.001 |

Table S3. Slope values for Secondary plots of the Lineweaver-Burk plot

| **C9 Concentration uM** | **Mean** | **SD** |
| --- | --- | --- |
| 60.50 | 1.374 | 0.061 |
| 30.25 | 0.942 | 0.048 |
| 15.13 | 0.649 | 0.043 |
| 0.00 | 0.203 | 0.019 |
